# Regulation of global gene expression in brain by TMP21

**DOI:** 10.1186/s13041-019-0460-5

**Published:** 2019-04-29

**Authors:** Xiaojie Zhang, Yili Wu, Fang Cai, Weihong Song

**Affiliations:** 1 Department of Psychiatry, The Second Xiangya Hospital, Central South University, Changsha, Hunan China; 2 National Clinical Research Center for Mental Disorders, Changsha, Hunan China; 3 Townsend Family Laboratories, Department of Psychiatry, The University of British Columbia, 2255 Wesbrook Mall, Vancouver, BC V6T 1Z3 Canada; 4 Shandong Collaborative Innovation Center for Diagnosis, Treatment and Behavioral Interventions of Mental Disorders, Institute of Mental Health, Jining Medical University, Jining, Shandong China

**Keywords:** TMP21, Gene expression profiling, Brain function, Alzheimer’s disease

## Abstract

TMP21, a type I transmembrane protein of thep24 protein family, mediates protein trafficking and maturation. Dysregulation of TMP21 is implicated in the pathogenesis of Alzheimer’s disease (AD). However, underlying mechanisms remain elusive. To reveal the function of TMP21 in the brain and the pathogenic role of TMP21 in the brain of AD, the global gene expression was profiled in the brain of TMP21 knockdown mice. We found that 8196 and 8195 genes are significantly altered in the hippocampus and cortex, respectively. The genes are involved in a number of brain function-related pathways, including glutamatergic synapse pathway, serotonergic synapse pathway, synaptic vesicle pathway, and long-term depression pathway. Moreover, the network analysis suggests that the TMP21 may contribute to the pathogenesis of AD by regulatingPI3K/Akt/GSK3β signalling pathway. Our study provides an insight into the physiological function of TMP21 in the brain and pathological role of TMP21 in AD.

## Introduction

TMP21 (Transmembrane protein, 21KD), also known as TMED10 (Transmembrane emp24 domain-containing protein 10), p23 or p24d1, is a type I transmembrane protein belonging to the p24 family [1]. TMP21 is ubiquitously expressed in the brain, heart, liver, lung, pancreas etc., and is critical for the maintenance of physiological functions [2]. TMP21 knockout mice are embryonic lethal, while TMP21 transgenic mice with a sustained high level of TMP21 expression display complex neurological problems and early death [3, 4]. The human TMP21 gene is located on Chromosome14q24.3, spanning 45,179 bp genomic DNA and including 5 exons and 4 introns (Robert Blum et al., 1996). Its gene expression is transcriptionally regulated by NFAT signalling [5]. TMP21 consists of 219 amino acids and is a conserved vesicle trafficking protein in eukaryotes from yeast to mammals [6–9]. TMP21 not only contributes to the generation and maintenance of Golgi apparatus and cis Glogi network (CGN), but also plays a critical role in protein transport by mediating both anterograde and retrograde transport between endoplasmic reticulum (ER) and Golgi apparatus [3, 10–12]. It co-partitions with GPI-anchored proteins, facilitating their ER export and guides them into biosynthetically early raft-like structures [13].

Alzheimer’s disease (AD), the most common form of dementia in the elderly [14] and growing evidence indicates that TMP21 is dysregulated in AD. The reduced level of TMP21 was detected in the frontal cortex and hippocampus of AD brains [15]. The transcription of TMP21 is regulated by calcineurin-NFAT signalling which is dysregulated in AD, contributing to the cognitive impairment [5, 16, 17]. The regulator of calcineurin 1 (RCAN1) is significantly increased in AD brains, which inhibits calcineurin-NFAT signalling [18–22]. Moreover, the single nucleotide polymorphism (SNP) rs12435391 in intron 4 of the *TMP21* gene isassociated with AD by accelerating TMP21 pre-mRNA splicing leading to increased expression of TMP21 [23]. Importantly, dysregulated TMP21 plays a pivotal role in the pathogenesis of AD. Deposition of Aβ to form neuritic plaques is the hallmark of AD neuropathology. Aβ is derived from APP by sequential cleavages of β- and γ-secretases [24–26].TMP21 was identified as a member of the γ-secretase complex to regulate APP processing to generate amyloid beta protein (Aβ) [27]. As the regulator of γ-secretase, dysregulated TMP21 contributes to increased Aβ generation and neuritic plaque formation in AD [23]. However, the role of TMP21 in brain function and in AD pathogenesis remains elusive.

To reveal the function of TMP21 in the brain and the pathogenic role of TMP21 in the brain of AD, the global gene expression was examined in the brain of TMP21 knockdown mice. The results showed that 8196 and 8195 genes involved in a number of brain function-related pathways are significantly altered in hippocampus and cortex, respectively. The network analysis suggests that the TMP21 may contribute to the pathogenesis of AD by regulating PI3K/Akt/GSK3β signalling pathway.

## Materials and methods

### Animals

Animal experiment protocols in this study were in accordance with guidelines established by the Canadian Council on Animal Care and approved by the University of British Columbia Animal Care Committee.S2P23 is the hemizygous TMP21knockout (TMP21^+/−^) mice by replacing the first exon of TMP21 with the neomycin resistance gene in a C57BL/6 background [3]. These S2P23 mice were bred in the Animal Research Unit (ARU) at the University of British Columbia Hospital. The ear punch biopsies were collected and digested in 300 μL lysis buffer (10 mM Tris HCl pH 8.0, 10 mM EDTA pH 8.0; 150 mM NaCl; 0.5% SDS) with 100 ng/ml proteinase K (New England Biolabs) overnight at 55 °C while rotating. DNA was purified using phenol/chloroform, precipitated with 0.7X volume of isopropanol, and dissolved in 50 μL TE buffer (pH 7.4).The PCR was performed by using forward primer G-TMP21mice-F (5′-ccggactctaggtccgccaa), and reverse primers G-TMP21mice-R (5′-tctggtttgtttggcccactctccg) and G-TMP21mice-Neo (5′-aattcgccaatgacaagacgct).The heterozygous S2P23 mice displayed two PCR-amplified DNA bands of 486bp and 260bp.

### Whole-genome gene expression assay

Hippocampus and cortex were dissected from 4(2 female and 2 male) wildtype and 4(1 female and 3 male) TMP21^+/−^mice at age of 2 months. RNA was isolated from mouse brain tissue using TRI-Reagent (Sigma-Aldrich). Thermoscript Reverse Transcription kit (Invitrogen) was used to synthesize the first strand cDNA following the manufacturer’s instruction. cRNA was amplified and purified by using Illumina Total Prep RNA amplification kit (Life Technologies) as described previously [28, 29]. 1.5 μgcRNA was used for whole-genome gene expression direct hybridization assay with mouse WG-6 v2.0 Expression Beadchip (Illumina) following the manufacturer’s instructions.

### Immunoblotting

Hippocampus and cortex were washed in ice-cold PBS and lysed by sonication with RIPA-DOC buffer containing 50 mM TrisHCl (pH 7.2), 150 mMNaCl, 1% deoxycholate, 2–3% Triton X-100, 0.1% SDS and protease inhibitor cocktail Complete (Roche). Protein lysates were diluted in 4XSDS-sample buffer and separated on 12% Tris-glycine SDS-PAGE geland transferred to polyvindylidine fluoride (PVDF-FL) membranes. Membranes were blocked in PBS containing 5% non-fat dried milk and incubated with the primary antibodies diluted in the blocking buffer at 4 °C overnight. Rabbit anti-TMP21 antibody T21 (1:1000) was generated by inoculating rabbit with synthetic peptide HKDLLVTGAYEIHK, this peptide shared 100% sequence homology with both mouse and human TMP21 [30]. Human p24a was detected by mouse monoclonal antibody TMED2 (1:2000) (C-8) (Santa Cruz Biotechnology). The antibody AC-15 (1:5000) (Abcam, Cambridge, MA, USA and Sigma) was used to detect β-actin. Then the membranes were rinsed in PBS-T and incubated with near-infrared fluorescence-labeled secondary antibodies IRDyeTM680-labeled goat anti-rabbit (1:100,000) and IRDyeTM800-labeled goat anti-mouse antibodies (1:100,000)(Lincoln, NE, USA)in PBS-T at room temperature for 1 h, after further rinsed in PBS-T the membrane was scanned by LI-COR Odyssey R system.

### Statistical analysis

Student’s *t* test was performed for the quantification of immunoblotting. Values of *P* < 0.05 were considered significant. Differential gene expression was analyzed by Beadstudio, Gene ontology (GO) analysis was performed on the Database for Annotation, Visualization, and Integrated Discovery (DAVID). Pathway analysis was performed on the basis of the Kyoto Encyclopedia of Genes and Genomes (KEGG) database to identify the significantly enriched pathways. The Fisher exact test was used to select the significant pathways [28, 29]. Signal-net analysis of gene–gene interaction network was constructed on the basis of the DEGs dataset [31, 32].

## Results

### Transcriptional profiling of S2P23 mouse hippocampus

**Fig. 1:**
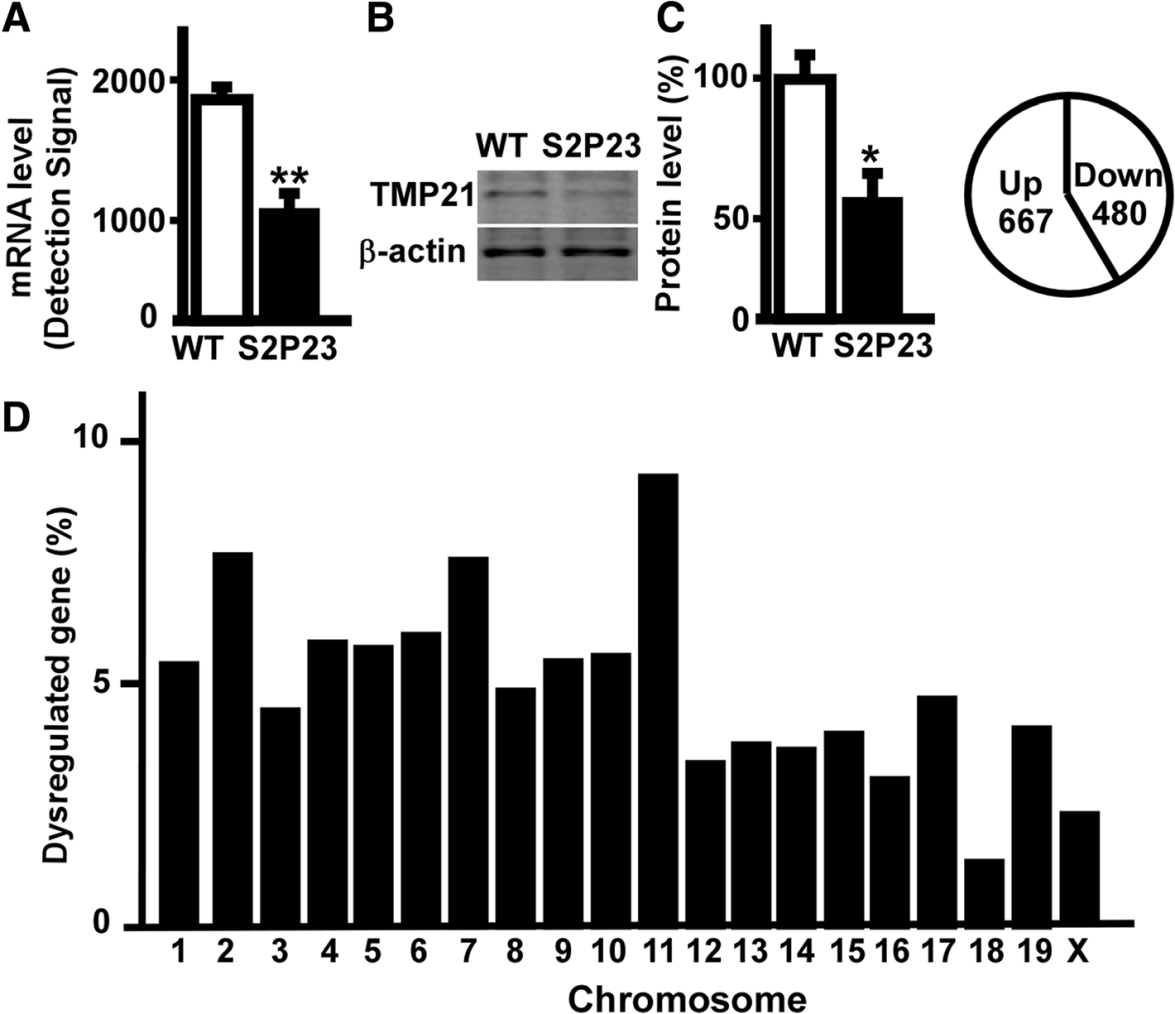
Transcriptional profiling of S2P23 mouse hippocampus. **a** Amplified RNA from the hippocampus of wild type mice and S2P23 mice was used to perform whole genome expression assay. The expression of TMP21 gene was significantly down-regulated. Values represent mean ± SEM; *n* = 3, *p* < 0.05by bead studio analysis. **b** Protein lysates from the hippocampus of wild type mice and S2P23 mice were resolved on 12% Tris-Glycine SDS-PAGE and endogenous TMP21was detected by T21 antibody. β-actin served as the internal control was detected by mouse anti-actin antibody. The values represent mean ± SEM; *n* = 3 each group, *p < 0.05 by Student’s t-test. **c** 1147genes were significantly regulated including667 upregulated genes and 480 downregulated genes. **d** Chromosome distribution of significantly altered genes in the hippocampus of S2P23 mouse brains. Values represent the percentage of differentially expressed genes on each chromosome at *p* < 0.05 and ± 1.50-fold cut-off

To investigate the role of TMP21 in the regulation of global gene expression, whole genome expression assay was performed in the hippocampus of S2P23 mice. The expression level of the *TMP 21* gene was reduced to 63.6% (Fig. 1a). Compared with wildtype mice, the expression of TMP21 protein was significantly down-regulated in the hippocampusof S2P23 mice (*p* = 0.022)(Fig.1b). The whole genome gene expression assay showed that 30,854 genes were detected with 45,281 illumina probes, and 8196 genes were significantly altered in the hippocampus of S2P23 mice at *p* < 0.05, including 3833 up-regulated genes and 4363 down-regulated genes. The normalization, background extraction and illumine custom false discovery rate correction were applied. The expression of 1542 genes was significantly changed at 1.5-fold cut-off. 1147 significantly regulated genes were counted when unidentified gene symbols and genes on unknown chromosome were ignored. Among them, 667 genes were significantly up-regulated and 480 genes were significantly down-regulated (Fig.1c).

Comparing to wildtype mice, we found that the differentially expressed genes in the hippocampus of S2P23 mice distributed on all 20 chromosomes, ranging from 16 genes on chromosome 18 to 108 genes on chromosome 11. There were 63, 89, 52, 68, 67, 70, 88, 57, 64, 65, 108, 40, 44, 43, 47, 36, 55, 16, 48 and 27 significantly dysregulated genes on chromosome 1to X, respectively. The percentage of differentially expressed genes on each chromosome was calculated as5.49, 7.76, 4.53, 5.93, 5.84, 6.10, 7.67, 4.97, 5.58, 5.67, 9.42, 3.49, 3.84, 3.75, 4.10, 3.14, 4.80, 1.40, 4.18 and 2.35% (Fig. 1d).

### Transcriptional profiling of S2P23 mouse cortex

**Fig. 2:**
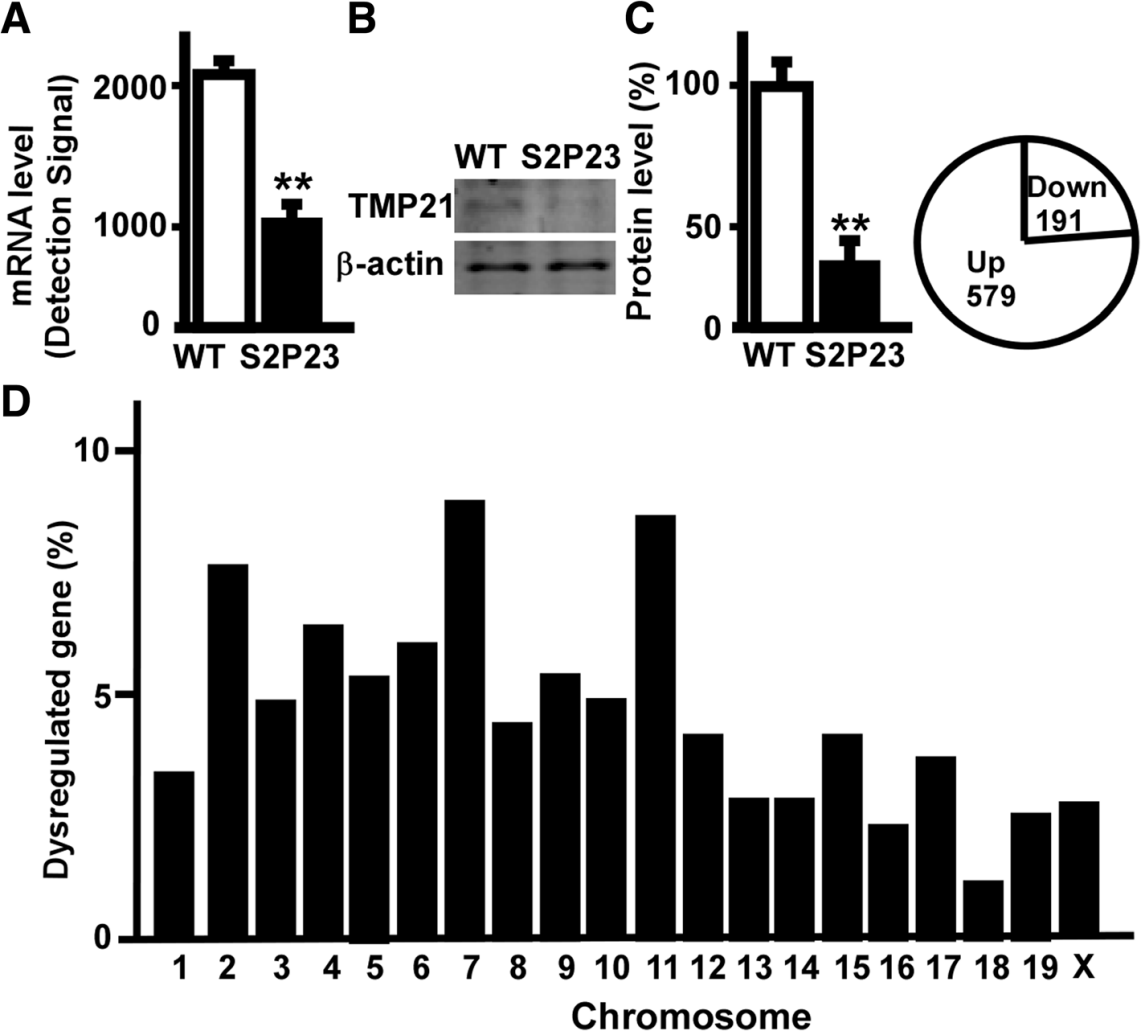
Transcriptional profiling of S2P23 mouse cortex. **a** Amplified RNA from the cortex of wild type mice and S2P23 mice was used to perform whole genome expression assay. The expression of TMP21 gene was significantly down-regulated. Values represent mean ± SEM; *n* = 3, *p* < 0.05by bead studio analysis. **b** Protein lysates from the cortex of wild type mice and S2P23 mice were resolved on 12% Tris-Glycine SDS-PAGE and endogenous TMP21 was detected by T21 antibody. β-actin served as the internal control was detected by mouse anti-actin antibody. The values represent mean ± SEM; *n* = 3 each group, *p < 0.05 by Student’s t-test. **c** 770 significantly regulated genes included 579 upregulated genes and 191 downregulated genes. **d** Chromosome distribution of significantly altered genes in the cortex of S2P23 mouse brains. Values represent the percentage of differentially expressed genes on each chromosome at *p* < 0.05 and ± 1.50-fold cut-off

To investigate the role of downregulated TMP21 in the regulation of global gene expression in the cortex, whole genome expression assay was performed in the cortex of S2P23 mice. The expression of the *TMP21* gene was reduced to 51.0% in the cortex (Fig. 2a). Compared with wildtype mice, the expression of TMP21 protein was significantly down-regulated in the cortex of S2P23 mice (*p* = 0.002)(Fig. 2a). The whole genome gene expression assay showed that 30,854 genes were detected with total 45,281 illumina probes, and 8195 genes were significantly altered in the cortex of S2P23 mice at *p* < 0.05, including 3329 up-regulated genes and 4866 down-regulated genes at *p* < 0.05. The normalization, background extraction and illumine custom false discovery rate correction were applied. The expression of 1004 genes in cortex were significantly changed at 1.5-fold cut-off. 770 significantly regulated genes were counted when unidentified gene symbols and genes on unknown chromosome were ignored. In total, 579 genes were significantly up-regulated and 191 genes were significantly down-regulated in cortex (Fig. 2c).

Comparing to wildtype mice, the differentially expressed genes distributed on all 20 chromosomes, ranging from 13 genes on chromosome 18 to 64 genes on chromosome 7. There were 33, 61, 38, 51, 42, 46, 64, 35, 42, 39, 62, 33, 29, 29, 37, 23, 35, 13, 28 and 30 significantly dysregulated genes on chromosome 1to X, respectively. The percentage of differentially expressed genes on each chromosome was calculated as4.29, 7.92, 4.94, 6.62, 5.45, 5.97, 8.31, 4.55, 5.45, 5.06, 8.05, 4.29, 3.77, 3.77, 4.81, 2.99, 4.55, 1.69, 3.64 and 3.90%.The data indicated that TMP21 has a wideeffect on gene transcription (Fig. 2d).

### Gene ontology and canonical pathway analysis of the dysregulated genes in the hippocampus

**Fig. 3:**
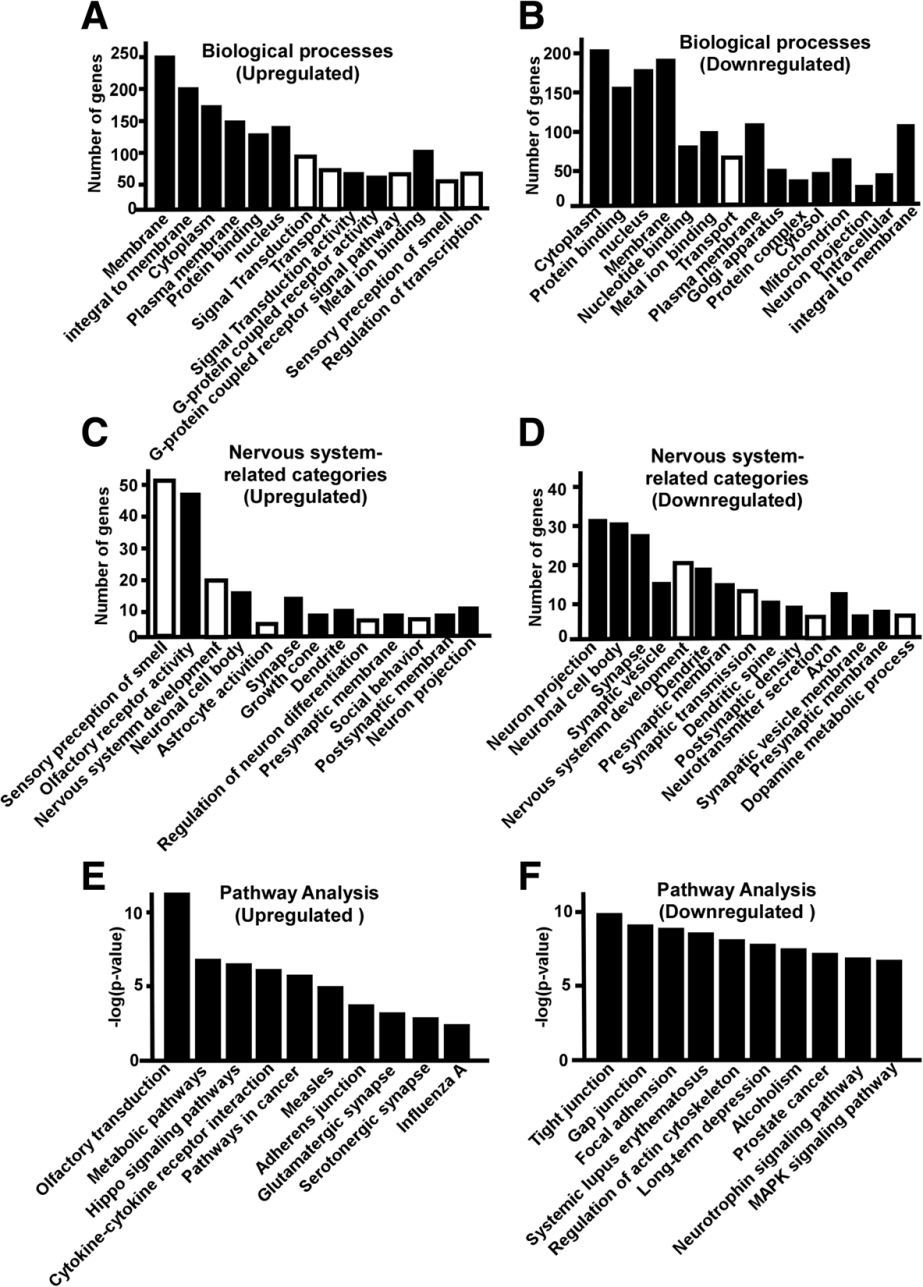
Gene Ontology and pathway analysis of the dysregulated genes in the hippocampus of S2P23 mice. **a** Significantly affect GO categories (Black bar) and subcategories (White bar) of biological process by upregulated genes are plotted, *p* < 0.05. **b** Significantly affect GO categories (Black bar) and subcategories (White bar) of biological process by downregulated genes are plotted, *p* < 0.05. **c** Nervous system related-Go categories affected by upregulated genes are plotted, *p* < 0.05. **d** Nervous system related-Go categories affected by downregulated genes are plotted, *p* < 0.05. **e** Pathways affected by upregulated genes are plotted, *p* < 0.05. **f** Pathways affected by downregulated genes are plotted, *p* < 0.05

To reveal the potential function of differentially expressed genes in the hippocampus of S2P23 mice, the enrichment analysis of gene ontology (GO) was performed. The upregulated genes were involved in 632GO categories (*p* < 0.05), including 379 GO categories of biological processes, 80 categories of cell component, and 173 GO categories of molecular function. The downregulated genes were involved in 710 categories (*p* < 0.05), including 398 GO categories of biological processes, 134 categories of cell component, and 178 GO categories of molecular function. The biological processes affected by the up-regulated genes included signal transduction (84 genes), transport (69 genes), G-protein coupled receptor signalling pathway (67 genes), sensory perception of smell (50 genes), regulation of transcription, DNA-dependent (65 genes), transcription, DNA-dependent (60 genes), response to stimulus (47 genes), ion transport (30 genes), metabolic process (35 genes) transmembrane transport (21 genes) etc. (Fig. 3a).On the other hand, 398 biological processes affected by the downregulated genes included transport (66 genes), regulation of transcription, DNA-dependent (57 genes),transcription, DNA-dependent (56 genes), nervous system development (18 genes), protein transport (25 genes) (Fig. 3b). 31 Go categories affected by upregulated genes were related to nervous systems, including sensory perception of smell (50 genes), olfactory receptor activity (48 genes), nervous system development (18 genes),neuronal cell body (16 genes), astrocyte activation (3 genes), synapse (15genes), growth cone (7 genes), dendrite (12 genes), regulation of neuron differentiation (4 genes) etc. (Fig. 3c).70 Go categories related to nervous systems were affected by downregulated genes, including neuron projection (28 genes), neuronal cell body (27 genes), synapse (25 genes), synapse vesicle (13 genes),nervous system development (20 genes),dendrite (19 genes), postsynaptic membrane (15 genes), synaptic transmission (12 genes), dendrite spine(10 genes), postsynaptic density(11 genes) etc. (Fig. 3d).

GO enrichment analysis only presents each function independently, while pathway analysis considers the functional dependency and molecular interaction [33, 34]. Thus, we performed pathway analysis by using KEGG. 53 pathways were significantly affected by upregulated genes (*p* < 0.05) in the hippocampus, including olfactory transduction, metabolic pathway, hippo signalling pathways, cytokine-cytokine receptor interaction, pathways in cancer, measles, adherence junction, glutamatergic synapse, serotonergic synapse, influenza A, NF-kappa B signalling pathway, neuroactive ligand-receptor interaction(Fig. 3e). 110 pathways were significantly affected by the downregulated genes (*p* < 0.05) in the hippocampus, including tight junction, gap junction, focal adhesion, systemic lupus erythematosus, regulation of actin cytoskeleton, long-term depression, alcoholism, prostate cancer, neurotrophin signalling pathway, MAPK signalling pathway, estrogen signalling pathway, vascular smooth muscle contraction, viral carcinogenesis, GnRH signalling pathway, leukocyte transendothelial migration(Fig. 3f).

### Gene ontology and canonical pathway analysis of the dysregulated genes in the cortex

**Fig. 4:**
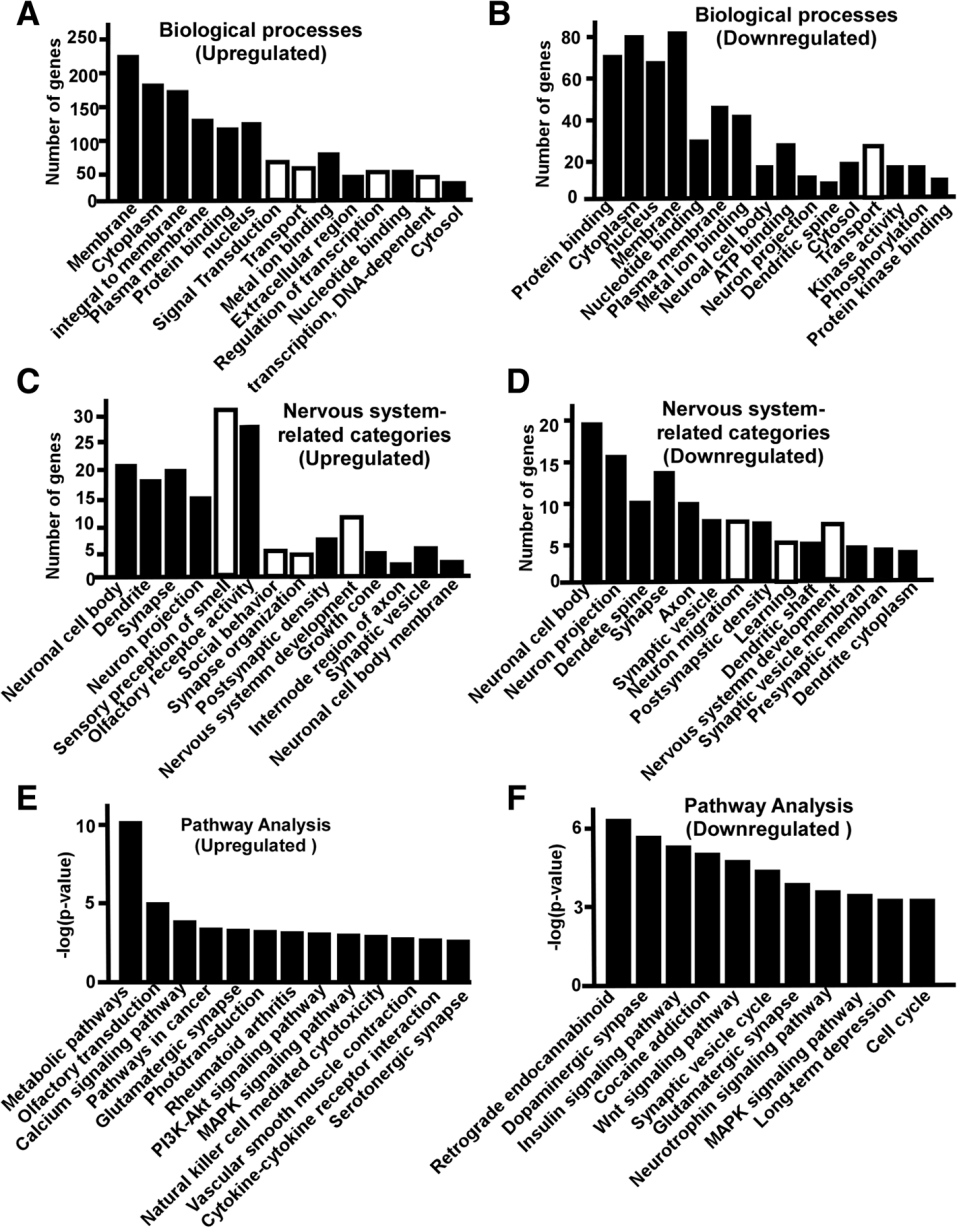
Gene Ontology and pathway analysis of the dysregulated genes in the cortex of S2P23 mice. **a** Significantly affect GO categories (Black bar) and subcategories (White bar) of biological process by upregulated genes are plotted, *p* < 0.05. **b** Significantly affect GO categories (Black bar) and subcategories (White bar) of biological process by downregulated genes are plotted, *p* < 0.05. **c** Nervous systemrelated-Go categories affected by upregulated genesare plotted, *p* < 0.05. **d** Nervous systemrelated-Go categories affected by downregulated genesare plotted, *p* < 0.05. **e** Pathways affected by upregulated genes are plotted, *p* < 0.05. **f** Pathways affected by downregulated genes are plotted, *p* < 0.05.

In the cortex of S2P23 mice, upregulated genes involved in 610 GO categories(*p* < 0.05), including 347 GO categories of biological processes, 90 categories of cell component, and 173 GO categories of molecular function. The downregulated genes involved in 498 GO categories, including 285 GO categories of biological processes, 85 categories of cell component, and 128 GO categories of molecular function. (*p* < 0.05). 347 Go categories of biological processes affected by upregulated genes included signal transduction (66 genes), transport (57 genes), regulation of transcription, DNA-dependent (55 genes), transcription, DNA-dependent (51 genes), multicellular organism development (35 genes), potassium ion transport (14 genes), G-protein coupled receptor signalling pathway (49 genes), ion transport (23 genes) etc. (Fig. 4a). On the other hand, the downregulated genes were involved in 285 biological processes (*p* < 0.05), including transport (29 genes), phosphorylation (19 genes), protein phosphorylation (17 genes), regulation of transcription, DNA-dependent (26 genes),negative regulation of apoptotic process (13 genes), neuron migration (7 genes), ion transport (13 genes),learning (5 genes) and transcription, DNA-dependent (22 genes) (Fig. 4b).27 Go categories affected by upregulated genes were related to nervous systems, including neuronal cell body (20 genes), dendrite (17 genes), synapse (19 genes), neuron projection (15 genes), sensory perception of smell (30 genes), olfactory receptor activity (28 genes), social behavior (5genes),synapse organization (4 genes), postsynaptic density (7 genes), nervous system development(11 genes) etc. (Fig. 4c). 70 Go categories affected by downregulated genes were related to nervous systems, including neuronal cell body (18 genes), neuron projection (15 genes), dendrite spine (10 genes), synapse (13 genes), axon (10 genes), synaptic vesicle (7 genes), neuron migration (7 genes), postsynaptic density (7 genes), learning (5 genes), dendrite shaft (5 genes), nervous system development (9 genes) (Fig. 4d).

Sixty-one pathways were significantly affected by upregulated genes (*p* < 0.05) in the cortex, including metabolic pathway, olfactory transduction, calcium signaling pathway, pathways in cancer, glutamatergic synapse, phototransduction, rheumatoid arthritis, PI3K-Akt signaling pathway, MAPK signalling pathway, natural killer cell mediated cytotoxicity, vascular smooth muscle contraction, cytokine-cytokine receptor interaction, serotonergic synapse, morphine addiction etc.(Fig. 4e). 52 pathways were significantly affected by downregulated genes in the cortex (*p* < 0.05), including retrograde endocannabinoid signalling pathway, dopaminergic synapse, insulin signalling pathway, cocaine addiction, Wnt signaling pathway, synaptic vesicle cycle, glutamatergic synapse, MAPK signalling pathway, long-term depression, cell cycle, amphetamine addiction, adipocytokine signalling pathway, Epstein-Barr virus infection, circadian rhythm, progesterone-mediated oocyte maturation, prostate cancer, protein processing in endoplasmic reticulum(Fig. 4f).

### Network analysis of dysregulated genes in the hippocampus and cortex

**Fig. 5:**
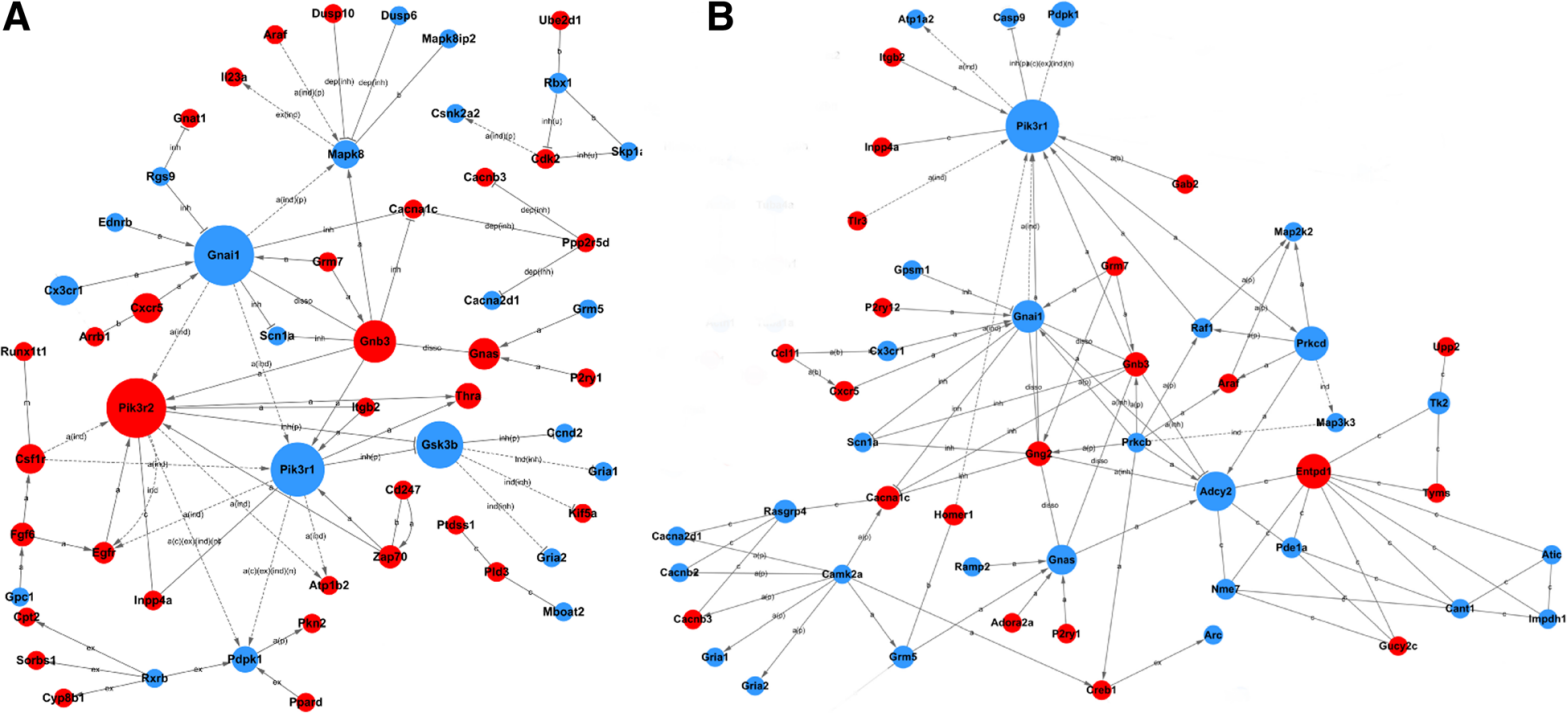
Gene-gene interaction networks. **a** Gene-gene interaction networks were generated based on the dysregulated genes in the hippocampus by using Gene Signal Network software. **b** Gene-gene interaction networkswere generated based on the dysregulated genes in the cortex. Red and green colored genes represent up- and down-regulated genes, respectively. Letter a represents activation, b represents binding/associated, c represents compound, inh represents inhibition, ind represents indirect effect, u represents ubiquitination, m represents missing interaction, ex represents expression, p represents phosphorylation, rep represents repression, and disso represents dissociation

The gene signal network analysis acquires interaction between genes in single pathway. The networks were generated from top ranked differentially expressed genes, including eight and seven networks in the hippocampus and cortex, respectively. The top eight dysregulated genes in the center of gene signal network in hippocampus were phosphoinositide-3-kinase regulatory subunit 1 (PiK3r1), adenylate cyclase 2 (Adcy2), ectonucleoside triphosphate diphosphohydrolase 1 (Entpd1), protein kinase C, delta (Prkcd), guanine nucleotide binding protein (G protein), alpha inhibiting 1 (Gnai1), guanine nucleotide binding protein, alpha stimulating complex locus (Gnas), 3-phosphoinositide dependent protein kinase 1 (Pdpk1) and FMS-like tyrosine kinase 1(Flt1) (Fig. 5a). The top eight dysregulated genes in the center of gene signal network in cortex were guanine nucleotide binding protein (G protein), alpha inhibiting 1(Gnai1), phosphoinositide-3-kinase regulatory subunit 2(PiK3r2), phosphoinositide-3-kinase regulatory subunit 1(PiK3r1), glycogen synthase kinase 3 beta (GSK3β), guanine nucleotide binding protein (G protein), beta 3(Gnb3), guanine nucleotide binding protein, alpha stimulating complex locus (Gnas), chemokine (C-X-C motif) receptor 5(Cxcr5) and chemokine (C-X3-C) receptor 1 (Cx3cr1) (Fig. 5b). Many common genes, dysregulated in both hippocampus and cortex, were involved in the networks with minor difference. For example, the expression of PiK3r1, Gnai1and GSK3β were significantly downregulated in both hippocampus and cortex of S2P23 mice, while Gnas was significantly downregulated in hippocampus but upregulated in cortex. PiK3r1 was a hub gene in the represented networks of both hippocampus and cortex (Fig. 5a, b), while GSK3βwas a key node in the represented networks of the cortex (Fig. 5b).

## Discussion

TMP21is highly expressed in the brain during the embryonic development stage, however, it declines with age after birth [3, 15]. Complete TMP21 knockout is embryonic lethal in mice [3]. Patients with AD show a reduced level of TMP21 in the frontal cortex and hippocampus, but not in the cerebellum [15].To investigate the physiological function and pathological role of TMP21 in the cortex and hippocampus, the gene expression profiling was performed inTMP21 hemizygous mouse strain, S2P23mice [3]. 8196 genes and 8195 genes, around 18% of total 45,281 illumina genes were significantly altered in the hippocampus and cortexof S2P23 mice, respectively. The affected genes distribute on 20 chromosomes, ranging from 1.39 to 9.42% in the hippocampus and from 1.69 to 8.31% in the cortex. The data indicate that TMP21 has global effect on gene transcription and covering all chromosomes, not limiting in small numbers of genes or specific chromosome. GO enrichment analysis showed that the processes involved in membrane, cytoplasma, protein binding and transport were significantly affected by the downregulation of TMP21 in both hippocampus and cortex of S2P23 mice. Pathway analysis further supported the results from GO enrichment analysis. For example, tight junction, gap junction and adherence junction are all involved in membrane and integral to membrane.

As a regulator of the γ-secretase complex, TMP21 selectively influences γ-site cleavage on APP but has no effect on ε-site cleavage of APP or Notch [27, 35]. It suggests that TMP21 could be a potential therapeutic target to specifically reduce Aβ generation without affecting Notch signalling pathway. Similar to other members of γ-secretase complex, TMP21 can be degraded by the ubiquitin-proteasome pathway [30]. Interestingly, it has been reported that TMP21did not exist in the mature and active γ-secretase complex [36]. Consistently, increased immature Nicastrin (NCT) was detected whenTMP21 was knockdown to approximately 20%. Our pathway analysis showed that AD-related pathway was significantly affected by the downregulated genes. It indicated that the dysregulated expression of TMP21might play a pivotal role in AD pathogenesis by regulating the expression of a number of genes in addition to modulate γ-secretase activity.

Phosphoinositide-3-kinase regulatory subunit 1(PiK3r1) is the key subunit of phosphoinositide-3-kinase (PI3K) which is implicated in AD pathogenesis by affecting PI3K/AKT/glycogen synthase kinase 3β (GSK3β) signalling [37, 38]. In this study, we showed that it was a hub gene of the networks, which was significantly downregulated in both hippocampus and cortex of S2P23 mice. In addition, GSK3β, a key node of the networks, was significantly regulated, which has been showed to play a critical role in the pathogenesis of Alzheimer’s disease by promoting Aβ generation and Tau hyperphosphorylation [39]. It indicates that the effect of TMP21on PI3K/Akt/GSK3β signalling pathway might be another key mechanism of promoting Aβ generation and Tau hyperphosphorylation. Moreover, TMP21could affect AD pathogenesis via altering epigenetic regulation. For example, PI3K/Akt pathway is involved in regulating the function of histone deacetylase 4 (HDAC4), while HDAC4 plays a pivotal role in various neurological diseases including AD [40–42]. These evidence suggests that dysregulation of TMP21 might play a complex role in the pathogenesis of AD in addition to regulating protein trafficking and γ-secretase activity.

The effect of deficient TMP21 on gene expression might be linked to the secondary effects of TMP21on early embryonic development and protein trafficking as TMP21 per se might not directly regulate gene transcription. Firstly, homozygous deletion of TMP21 results in embryonic lethality at very early stage and no homozygous embryo can be produced, indicating the fundamental role of TMP21 in the early embryonic development of mammals [3]. Even though the heterozygous TMP21 mice was alive and grossly normal, inactivation of one allele not only led to reduced level of TMP21 but also led to reduced levels of other members of the p24 family. The reduction in steady-state protein levels might lead to the alteration of gene expression. Secondly, as a member of vesicular trafficking protein family, TMP21 mediates the protein ER/ERGIC/Golgi trafficking and protein transport [12, 43–45]. TMP21 might greatly affect functions of proteins that mature through the early constitutive secretory pathway from the endoplasmic reticulum (ER) to Golgi complex. It is possible that TMP21 deficiency affects the maturation and normal function of various proteins implicated in the regulation of gene transcription, contributing to the dysregulation of gene transcription.

## Abbreviations

AD: Alzheimer’s disease
APP: Amyloid-β precursor protein
Aβ: β; amyloid
GO: Gene ontology
SNP: Single nucleotide polymorphism
TMED10: Transmembrane emp24 domain-containing protein 10
TMP21: Transmembrane protein, 21KD
